# Differences in autophagy marker levels at birth in preterm vs. term infants

**DOI:** 10.1038/s41390-024-03273-6

**Published:** 2024-05-29

**Authors:** Noëmi Künstle, Olga Gorlanova, Andrea Marten, Loretta Müller, Pawan Sharma, Martin Röösli, Pablo Sinues, Primo Schär, David Schürmann, Céline Rüttimann, Carla Rebeca Da Silva Sena, Uri Nahum, Jakob Usemann, Ruth Steinberg, Sophie Yammine, Sven Schulzke, Philipp Latzin, Urs Frey, on behalf of the BILD study group, Noëmi Künstle, Olga Gorlanova, Andrea Marten, Loretta Müller, Martin Röösli, Pablo Sinues, Céline Rüttimann, Uri Nahum, Jakob Usemann, Ruth Steinberg, Sophie Yammine, Sven Schulzke, Philipp Latzin, Urs Frey, Fiona Beck, Xenia Bovermann, Carmen Casaulta, Marion Curdy, Carla Rebeca Da Silva Sena, Kees de Hoogh, Bettina Frauchiger, Léa Kim-Mi Ho Dac, Elisabeth Kieninger, Insa Korten, Marc-Alexander Oestreich, Benjamin Stöcklin, Carmen Streibel, Florian Wyler

**Affiliations:** 1 University Children’s Hospital Basel UKBB, University of Basel, Basel, Switzerland; 2 Division of Pediatric Respiratory Medicine and Allergology, Department of Pediatrics, Inselspital, Bern University Hospital, University of Bern, Bern, Switzerland; 3 Center for Translational Medicine, Division of Pulmonary, Allergy and Critical Care Medicine, Jane & Leonard Korman Respiratory Institute, Sidney Kimmel Medical College, Thomas Jefferson University, Philadelphia, PA USA; 4 Swiss Tropical and Public Health Institute, Allschwil, Switzerland and University of Basel, Basel, Switzerland; 5 Department of Biomedical Engineering, University of Basel, Allschwil, Switzerland; 6 Department of Biomedicine, University of Basel, Basel, Switzerland; 7 Priority Research Centre GrowUpWell® and Hunter Medical Research Institute, University of Newcastle, Newcastle, NSW Australia; 8 Institute for Medical Engineering and Medical Informatics, University of Applied Sciences and Arts Northwestern Switzerland, Muttenz, Switzerland

## Abstract

**Background:**

Preterm infants are susceptible to oxidative stress and prone to respiratory diseases. Autophagy is an important defense mechanism against oxidative-stress-induced cell damage and involved in lung development and respiratory morbidity. We hypothesized that autophagy marker levels differ between preterm and term infants.

**Methods:**

In the prospective Basel-Bern Infant Lung Development (BILD) birth cohort we compared cord blood levels of macroautophagy (Beclin-1, LC3B), selective autophagy (p62) and regulation of autophagy (SIRT1) in 64 preterm and 453 term infants.

**Results:**

Beclin-1 and LC3B did not differ between preterm and term infants. However, p62 was higher (0.37, 95% confidence interval (CI) 0.05;0.69 in log2-transformed level, *p* = 0.025, *p*_adj_ = 0.050) and SIRT1 lower in preterm infants (−0.55, 95% CI −0.78;−0.31 in log2-transformed level, *p*_adj_ < 0.001). Furthermore, p62 decreased (*p*_adj_-value for smoothing function was 0.018) and SIRT1 increased (0.10, 95% CI 0.07;0.13 in log2-transformed level, *p*_adj_ < 0.001) with increasing gestational age.

**Conclusion:**

Our findings suggest differential levels of key autophagy markers between preterm and term infants. This adds to the knowledge of the sparsely studied field of autophagy mechanisms in preterm infants and might be linked to impaired oxidative stress response, preterm birth, impaired lung development and higher susceptibility to respiratory morbidity in preterm infants.

**Impact:**

- To the best of our knowledge, this is the first study to investigate autophagy marker levels between human preterm and term infants in a large population-based sample in cord blood plasma
- This study demonstrates differential levels of key autophagy markers in preterm compared to term infants and an association with gestational age
- This may be linked to impaired oxidative stress response or developmental aspects and provide bases for future studies investigating the association with respiratory morbidity

## Introduction

Autophagy is a physiological process which plays a major role in degradation and recycling of damaged proteins or organelles and contributes to the maintenance of cellular homeostasis.^1^ Inducers of autophagy are mainly cellular stressors such as starvation, organelle damage, intracellular pathogens or reactive oxygen species (ROS).^2^ There is growing evidence that autophagy plays a critical role in embryonic development^3^ and is activated differently throughout the stages of organ development (e.g., of the lung^4^). Furthermore, it has been proposed that autophagy is involved in the development and maintenance of a healthy pregnancy^5^ and in mechanisms related to maternal birth induction.^6,7^ Accordingly, dysregulations in autophagy can result in pregnancy complications such as intrauterine growth retardation (IUGR), preeclampsia, gestational diabetes or preterm birth.^5,6^ A balance of autophagy is essential and both increase or decrease of autophagy have been linked to various diseases from neonatal hypoxic-ischemic encephalopathy^8^ to heart disorders or cancer.^9^

Cellular autophagy is significantly involved in the defense against oxidative stress because it helps cells to decrease oxidative damage.^10^ Excessive ROS generation, which leads to oxidative stress, is described to be associated with preterm birth^11^ and respiratory morbidity in preterm infants.^12^ The interaction between oxidative stress and autophagy is also of high importance in the pathogenesis of lung diseases throughout life (e.g., asthma and chronic obstructive lung disease).^10^ Moreover, it is known that preterm infants are particularly susceptible to oxidative stress shortly after birth, which makes them vulnerable to oxidative damage.^13^ This can lead to diseases such as respiratory distress syndrome or bronchopulmonary dysplasia.^13^ Our study group demonstrated this susceptibility by observing higher levels of fractional exhaled nitric oxide (a marker of inflammatory/oxidative stress response) in preterm infants compared to term infants in the context of air pollution.^14^

A deeper understanding of autophagy-related mechanisms is of great importance since approximately 10% of all infants are born prematurely,^15^ increasing the risk of short- and long-term morbidities in general^16^ and respiratory morbidity in particular.^17^ Taken together, the current literature suggests a significant involvement of autophagy in intra-uterine development, maintenance of a healthy pregnancy, preterm birth, oxidative stress response and respiratory outcome in preterm infants. However, there is limited evidence of autophagy processes in preterm human infants. Therefore, the objective of this study was to evaluate the differences in autophagy between preterm and term infants. We assessed several autophagy-related markers in cord blood. Beclin-1 is involved in autophagosome formation whereas microtubule-associated proteins 1A/1B light chain 3B (LC3B) participate in elongation and maturation of the autophagosome.^18^ During selective autophagy, the polyubiquitin-binding protein sequestosome 1 (p62/SQSTM1, hereafter p62) recruits ubiquitinated cargo (e.g., defective proteins) to the autophagosomes and binds to LC3B.^19^ p62 itself is degraded by autophagy and accumulates when autophagy is decreased.^20^ Sirtuin 1 (SIRT1) is a NAD^+^-dependent deacetylase and involved in different regulatory steps in the autophagy process, e.g., by deacetylation of LC3B^21^ and promotion of autophagy.^22^

We hypothesized that autophagy marker levels differ between preterm and term infants. To provide a basis for understanding autophagy in preterm infants, the aims of this study were: (i) to compare levels of autophagy markers in cord blood between preterm and term infants after adjusting for risk factors, (ii) to assess the association between gestational age and autophagy marker levels, and (iii) to evaluate the correlations of autophagy markers in preterm and term infants.

## Methods

### Study design and population

The Basel-Bern Infant Lung Development (BILD) birth cohort is a prospective study established in 1999 in Bern and 2011 in Basel, Switzerland (www.bild-cohort.ch).^23,24^ Recruitment of infants took place prenatally or shortly after birth and exclusion criteria were severe disease of mother or infant, maternal drug abuse other than smoking and need for ventilation for >3 days in term infants. Further details about the study protocol have been previously described.^23,24^ For this study, since analysis of autophagy markers was performed in 2019, infants born between April 1999 and February 2019 were included. Additional exclusion criteria for this study were unavailable cord blood plasma (e.g., due to collapsing umbilical cord or storage by the parents for other purposes), cord blood donation due to differences in sampling procedure, missing clinical or sociodemographic data and time from blood collection to processing (e.g., centrifugation) >3 days or insufficiently documented sampling procedure. This resulted in a study population of 64 preterm and 453 term infants.

Written informed consent was obtained from the parents. The study was approved by the Ethics Committee of Northwestern and Central Switzerland (EKNZ, Basel, Switzerland) and the Bernese Cantonal Ethics Research Committee (KEK, Bern, Switzerland).

### Risk factors and exposure

Information on possible risk factors was taken from birth records and interviews with standardized questionnaires. The most important risk factors were small for gestational age (SGA, defined as birth weight below 10^th^ percentile), mode of delivery (Caesarean section/vaginal delivery), sex (male/female), birth order (non-firstborn/firstborn) and maternal smoking during pregnancy (yes/no). Since the clinical assessment of IUGR is observer-dependent, SGA was used as a quantitative, well-defined proxy. Further risk factors were antibiotic use during the last twelve weeks of pregnancy (yes/no, available in *n* = 472) and chorioamnionitis (yes/no, available in *n* = 504), both as proxy for infections, and Apgar score at 5 minutes as proxy for hypoxia (available in *n* = 516). Information on preeclampsia and gestational diabetes was only available for a subgroup of infants (*n* = 111). Z-scores and percentiles for birth weight were calculated with the Fenton growth chart.^25^ Preterm birth was defined as <37 weeks of gestational age (GA) and infants were grouped into four groups (<28, 28 + 0 to 31 + 6, 32 + 0 to 36 + 6 and >37 + 0 weeks of GA) for visualization.

### Outcome: autophagy markers

In cord blood plasma we analyzed the autophagy markers Beclin-1, LC3B, p62 and SIRT1.^26–28^ The blood was centrifuged and stored at −80 °C until further analysis . The analysis was performed in plasma with ELISA Kits (AVIVA Systems Biology, San Diego, CA) for Beclin-1, LC3B and SIRT1 and ELISA Kits (ENZO Life Sciences, Farmingdale, NY) for p62. The manufacturer’s protocol was strictly followed, and detection limits reported by the manufacturer were used for each marker. Further details can be found in the Supplementary Material.

### Statistical analysis

To examine differences in characteristics between preterm and term infants, we compared the groups using the Mann–Whitney U test for continuous variables and Pearson’s χ^2^ test or Fisher’s exact test for categorical variables.

#### First aim

We compared autophagy marker levels between preterm and term infants using Tobit regression models to account for the left-censoring because of data below the limit of detection. Firstly, this was done in partially adjusted models (center adjusted (Basel/Bern)). Secondly, the models were adjusted for risk factors selected according to the literature search^29–34^ and previous analyses in our cohort.^35^ These were SGA, mode of delivery, sex, birth order, maternal smoking during pregnancy and study center. Furthermore, we adjusted for time from blood collection to processing and for temporary fridge storage until processing, since the samples were sensitive to time and temperature (e.g., Beclin-1 and LC3B tended to decrease and p62 and SIRT to increase depending on time from blood collection to processing). The adjusted model is considered as the main model. Due to right-skewed distribution, autophagy marker data were log2-transformed after adding one to all values.^36^

#### Second aim

The association between gestational age and autophagy marker levels was examined by Tobit regression models for Beclin-1, LC3B and SIRT1. Due to the non-linear relationship between gestational age and p62, a generalized additive Tobit model (GAM) was used with smoothing function for gestational age, using the *cenGAM* package. All models were adjusted for the above-mentioned risk factors. For visualization of gestational age, infants were presented in the four a priori defined groups.

#### Third aim

Correlations between cord blood markers were evaluated with Spearman’s correlation coefficient. We considered only markers whose biological relationships are described in the literature (Beclin-1 and LC3B, p62 and LC3B, SIRT1 and LC3B).

#### Sensitivity analysis

We additionally adjusted the main model for preeclampsia, gestational diabetes, antibiotic use during the last twelve weeks of pregnancy and chorioamnionitis separately. Antibiotic use was only tested in a sensitivity analysis because of missing data. Chorioamnionitis was not included from the beginning since it occurred mainly in preterm infants. We considered adjusting for Apgar score at 5 minutes. However, since the score is directly associated with prematurity^37^ and potentially also on the same pathway which leads to changes in autophagy marker levels, additional adjustment for it would result in overadjustment.^38^ Furthermore, we reran the main model after exclusion of infants with p62 levels below the detection limit. Additionally, we stratified the analysis by sex and further included a multiplicative interaction term between prematurity and sex into the main model. Additional sensitivity analyses including further investigations of autophagy markers in plasma and storage duration can be found in the Supplementary Material.

*P*-values were adjusted for multiple-testing using the Benjamini–Hochberg method. *P*-values and *p*_adj_-values < 0.05 were considered significant.

Data analysis was performed in Stata 16 (StataCorp, College Station, TX) and R version 4.3.2 (R Foundation, Vienna, Austria).

## Results

### Descriptive statistics

We included 517 infants in this study with complete data for autophagy markers, clinical and sociodemographic data (Supplementary Fig. S1). Population characteristics are listed in Table 1. The included population consisted of 64 preterm (12%, mean GA 33.33 weeks) and 453 term infants (88%, mean GA 39.78 weeks). Preterm infants were predominantly born in Basel, by Caesarean section, with a lower Apgar score at 5 minutes than term infants and as the mother’s firstborn child. Compared to the mothers of term infants, the mothers of preterm infants more frequently received antibiotics during the last twelve weeks of pregnancy, had higher rates of chorioamnionitis and were older at birth. In the subgroup with availability of corresponding data, more mothers of preterm infants were diagnosed with preeclampsia than mothers of term infants.

**Table 1:** Study population characteristics.

	Total *n* = 517	Preterm infants *n* = 64	Term infants *n* = 453	*p*-value^a^
Gestational age at birth, weeks	38.98 (2.55)	33.33 (2.69)	39.78 (1.12)	<0.001
Sex, male	267 (52%)	35 (55%)	232 (51%)	0.603
Mode of delivery, Caesarean section	140 (27%)	52 (81%)	88 (19%)	<0.001
Weight at birth, z-score	−0.18 (0.84)	−0.27 (0.75)	−0.17 (0.85)	0.564
Small for gestational age	48 (9%)	7 (11%)	41 (9%)	0.626
Apgar, 5 minutes^b^	9.07 (0.98)	8.31 (1.32)	9.17 (0.87)	<0.001
Birth order, non-firstborn	265 (51%)	20 (31%)	245 (54%)	0.001
Twin or triplet^c^	31 (6%)	27 (42%)	4 (1%)	<0.001
Maternal smoking during pregnancy	27 (5%)	1 (2%)	26 (6%)	0.232
Antibiotics during the last 12 weeks of pregnancy^d^	52 (10%)	16 (25%)	36 (8%)	<0.001
Chorioamnionitis^e^	16 (3%)	11 (17%)	5 (1%)	<0.001
Preeclampsia^f^	10 (2%)	10 (16%)	0 (0%)	0.001
Gestational diabetes^f^	8 (2%)	4 (6%)	4 (1%)	1.000
Maternal age at birth, years	32.91 (4.33)	34.70 (4.62)	32.66 (4.23)	0.001
Study center, Basel	122 (24%)	57 (89%)	65 (14%)	<0.001

Data are presented as mean ± SD or *n* (%).

^a^*P*-values were obtained using the Mann–Whitney U test, Pearson’s χ^2^ test or Fisher’s exact test.

^b^Data available for *n* = 516 (preterm, *n* = 64; term, *n* = 452).

^c^Only considered if both/all siblings included in study.

^d^Data available for *n* = 472 (preterm, *n* = 62; term, *n* = 410).

^e^Data available for *n* = 504 (preterm, *n* = 62; term, *n* = 442).

^f^Data available for *n* = 111 (preterm, *n* = 57; term, *n* = 54).

Significant center differences in term infants were found in respect to Apgar score at 5 minutes and antibiotic use during pregnancy (Supplementary Table S1).

### Group comparison of preterm and term infants

Autophagy marker levels and detection rates are shown in Table 2 and in Supplementary Table S2.

**Table 2:** Cord blood autophagy marker levels and detection rates.

		Preterm		Term	
	Detection limit	Median (IQR)	Detection rate	Median (IQR)	Detection rate
Beclin-1, ng/ml	0.1	0.43 (0.33–0.63)	100%	0.51 (0.36–0.66)	100%
LC3B, ng/ml	0.078	1.41 (0.66–2.46)	98%	2.10 (1.19–2.92)	99%
p62, ng/ml	0.1	0.75 (0.29–2.53)	83%	0.25 (0.00–0.65)	64%
SIRT1, μg/ml	0.000032	0.80 (0.50–1.50)	97%	2.40 (1.40–3.80)	99%

*IQR* interquartile range.

In the risk factor adjusted model we observed higher levels of p62 (0.37, 95% confidence interval (CI) 0.05–0.69 in log2-transformed level, *p* = 0.025, *p*_adj_ = 0.050) and lower levels of SIRT1 (−0.55, 95% CI −0.78 to −0.31 in log2-transformed level, *p*_adj_ = 3.82 × 10^–5^) in preterm compared to term infants (Table 3). No differences were found in Beclin-1 and LC3B levels between preterm and term infants.

**Table 3:** Association between preterm birth and cord blood autophagy marker levels in comparison to term infants.

	Partially adjusted model (center adjusted)			Fully adjusted model^a^			
	Coef	95% CI	*p*-value	Coef	95% CI	*p*-value	Adj. *p*-value
Beclin-1, ng/ml	0.02	−0.04 to 0.08	0.492	0.03	−0.04 to 0.09	0.371	0.495
LC3B, ng/ml	−0.12	−0.31 to 0.08	0.256	−0.03	−0.24 to 0.18	0.785	0.785
p62, ng/ml	0.49	0.19 to 0.80	0.001	0.37	0.05 to 0.69	0.025	0.050
SIRT1, μg/ml	−0.49	−0.72 to −0.26	3.39 × 10^–5^	−0.55	−0.78 to −0.31	9.54 × 10^–6^	3.82 × 10^–5^

Tobit regression model was used. Coefficient is shown for preterm infants in comparison to term infants (reference group). Results are presented in log2-transformed level. Adjusted *p*-values (*n* = 4) were calculated using the Benjamini–Hochberg method.

*Coef* coefficient, *CI* confidence interval, *Adj.* adjusted.

^a^Adjusted for small for gestational age (SGA), mode of delivery, sex, birth order, maternal smoking during pregnancy, time until processing, temporary fridge storage and center.

### Autophagy and gestational age

**Fig. 1:**
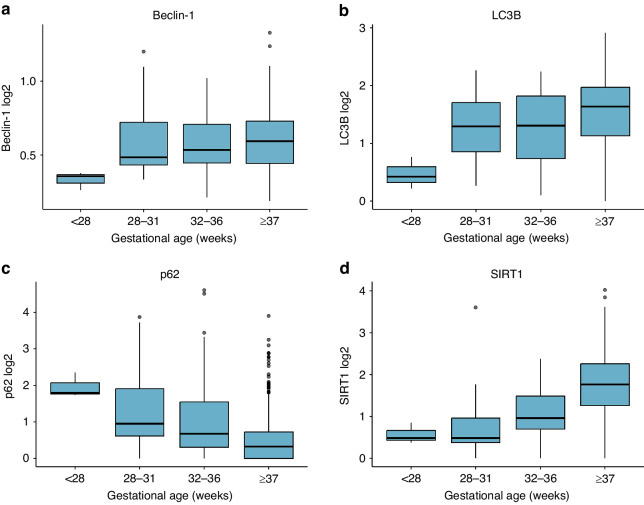
Autophagy marker levels at different gestational ages. **a** Beclin-1, **b** LC3B, **c** p62 and **d** SIRT1 marker levels are presented in log2-transformed level. Data are presented as median, lower and upper quartile, minimum and maximum data value and outliers.

**Fig. 2:**
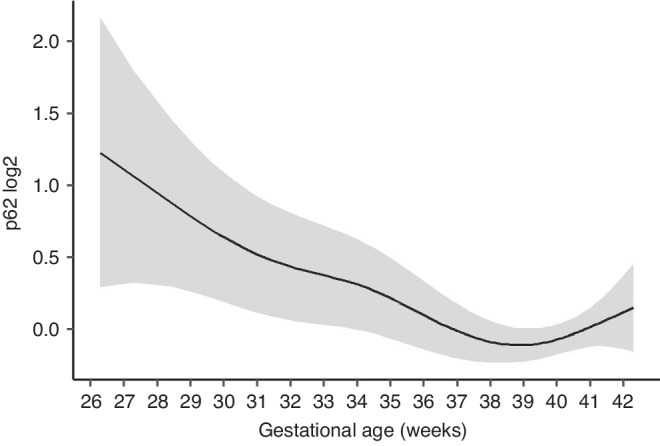
Association between gestational age and p62 level estimated using generalized additive Tobit model adjusted for small for gestational age (SGA), mode of delivery, sex, birth order, maternal smoking during pregnancy, time until processing, temporary fridge storage and center. p62 levels are presented in log2-transformed level. P_adj_-value for the smoothing function was 0.018. The gray area represents the 95% confidence interval. Adjusted p-values were calculated using the Benjamini–Hochberg method.

We observed increasing levels of SIRT1 (0.10, 95% CI 0.07–0.13 in log2-transformed level, *p*_adj_ = 2.23 × 10^–9^) per additional week of gestational age (Fig. 1d, Supplementary Table S3). The association between gestational age and p62 was significant (*p*_adj_-value for smoothing function was 0.018). Levels of p62 decreased until the 38^th^ week of gestation and increased slightly thereafter (Fig. 2).

### Correlations between autophagy markers

**Fig. 3:**
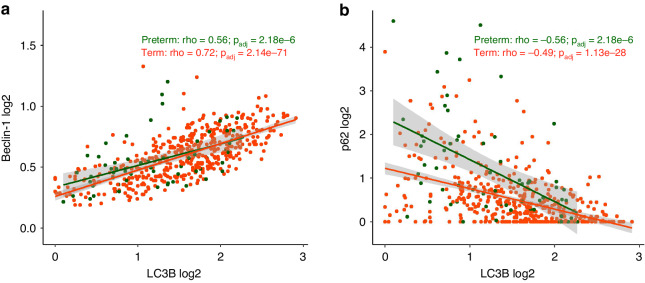
Comparison of associations between autophagy markers in preterm and term infants. Marker levels are presented in log2-transformed level, legend shows Spearman’s correlation coefficient (rho) with adjusted p-values using the Benjamini–Hochberg method. Association between (**a**) Beclin-1 and LC3B and between (**b**) p62 and LC3B are shown.

We observed a linear relationship between Beclin-1 and LC3B in both preterm and term infants (Fig. 3a). Furthermore, the correlation between p62 and LC3B was negative in both preterm and term infants (Fig. 3b) and was similar between these two groups (Supplementary Table S4).

### Sensitivity analysis

Levels of p62 remained higher and levels of SIRT1 lower in preterm compared to term infants after additional adjustment for preeclampsia, gestational diabetes, antibiotic use during the last twelve weeks of pregnancy and chorioamnionitis. Differences in p62 levels between preterm and term infants became even larger when additionally adjusting for these risk factors which have been separately added to the model for a subgroup of infants. The same was the case after exclusion of infants with p62 values below the detection limit. In contrast, differences in p62 levels between preterm and term infants were smaller and no longer statistically significant in the analysis stratified by sex, which could be due to smaller sample size. The interaction term between prematurity and sex was not significant. All sensitivity analysis results are shown in the Supplementary Material (Supplementary Tables S5–S14).

## Discussion

To the best of our knowledge, this is the first study that investigated autophagy marker levels between human preterm and term infants in a large population-based sample in cord blood plasma. We found higher levels of p62 and lower levels of SIRT1 in preterm infants in a risk factor adjusted analysis, although the difference in p62 levels was at the threshold for statistical significance after multiple comparison adjustment (*p* = 0.025, p_adj_ = 0.050). Furthermore, we observed increasing SIRT1 levels with increasing gestational age and decreasing p62 levels until the 38^th^ week of gestation as well as similar correlations between autophagy markers in preterm compared to term infants. Taken together, these findings support our hypothesis that preterm infants have differential levels of key autophagy markers compared to term infants.

The plausibility of our findings is supported by prior studies, which have focused on placental tissue analyses. Similarly to our observations, they found more p62 in preterm placental tissue,^33,34^ although Cao et al. observed the differences only between early preterm (<32 weeks of GA) and term placentas.^33^ Furthermore, consistent to our findings, Vassallo et al. reported lower SIRT1 in endothelial colony-forming cells (ECFCs) in the cord blood of preterm infants.^39^ Our data did not show evidence of differences in Beclin-1 and LC3B levels between preterm and term infants. This is consistent with Cao et al., who did not observe a difference in placental protein levels of Beclin-1^33^ and Akram et al., who described no difference in gene expression of Beclin-1 (*BECN1*) between preterm and term placental tissue, despite increased p62 protein levels in the preterm placentas.^34^ Conversely to our findings, Cao et al. observed lower levels of LC3-II in early preterm compared to term placental tissue.^33^ Although we have no clear explanation for this discrepancy, we suspect the different methodology to be the main reason. The described study was able to distinguish between LC3-I and LC3-II isoforms and they measured autophagy in placental tissue.

An increasing body of evidence suggests that autophagy plays an important role throughout pregnancy^5^ and that its activity correlates with gestational age.^39,40^ The findings of decreasing p62 in peripheral mononuclear cells incubated with the sera of pregnant women^40^ and increasing SIRT1 in ECFCs in cord blood^39^ with increasing gestational age adds support to our findings in cord blood plasma. The strong positive correlation between Beclin-1 and LC3B and negative correlation between p62 and LC3B in both preterm and term infants are consistent with literature which suggests that Beclin-1 and LC3B are increased and p62 decreased when autophagy is induced.^18,33^ These observations indicate that the findings are not random, but part of the autophagy pathway.

Although the mechanisms of release of intracellular markers into the plasma are not fully understood, the involvement of extracellular vesicles (EVs) has been identified, which can potentially help secrete autophagy markers from the cell.^41,42^ EVs are known to play a crucial role in cell–cell communication and have been implicated in various physiological and pathological processes including autophagy.^41,43^ Further evidence supporting the relationship between plasma and intracellular levels has been reported by Viana-Mattioli et al., where they experimentally demonstrated the influence of plasma with decreased SIRT1 levels on intracellular SIRT1 levels.^27^ Autophagy marker levels have already been measured in adult plasma and serum.^27,28,44,45^

Increased p62 levels can indicate impaired or downregulated autophagy activity, as p62 accumulates when autophagy is impaired.^20^ We have observed this in preterm infants, suggesting lower autophagy activity compared to term infants. Our observed decrease in SIRT1 levels supports the interpretation that autophagy is reduced in preterm infants, as SIRT1 is a promoter of autophagy and the knockdown of SIRT1 has been associated with decreased autophagy in cardiomyocytes.^22^ Additionally, both p62 and SIRT1 are involved in the removal of damaged mitochondria, whose accumulation can lead to oxidative stress.^19,46,47^ The absence of differences in Beclin-1 and LC3B levels between preterm and term infants leads us to hypothesize that the conditions for functional autophagy initiation are not disturbed in preterm infants. Instead, we suspect that altered mechanisms in the regulatory processes of autophagy (SIRT1) and completion of the autophagy process (accumulation of p62) are reasons for the differences between preterm and term infants.

Our observed association of gestational age with p62 and SIRT1 levels could indicate increasing nutrient need with progressing gestation,^7^ different developmental stages that require autophagy activation^4^ or increasing maturation of autophagy system. The observed increase of p62 levels after the 38^th^ week of gestation could be due to inhibition of autophagy near term to induce labor, as proposed for myometrial cells^7^ and could be a reason for the adjusted p-value at the threshold for statistical significance in the comparison of p62 levels between preterm and term infants.

There is currently a lack of evidence on how dysregulation of autophagy is involved in mechanisms of preterm birth. Previous studies have postulated that decreased SIRT1 is related to premature placental aging in preeclampsia^48^ and that oxidative-stress-induced damage of fetal membranes leads to preterm birth.^11^ Furthermore, a link between oxidative stress damage and premature aging of fetal cells has been made.^11^ Therefore, we hypothesize that premature aging and impaired ability of the fetus to defend against oxidative stress are linked to impaired autophagy in preterm infants and contribute to preterm birth. Defective oxidative stress response has also been implicated in impaired lung development and respiratory morbidity,^12^ which is a major problem in preterm infants.^17^ Autophagy plays a role in respiratory morbidity^10^ and animal models provide first evidence that autophagy is already involved in lung development.^4^ Furthermore, it is involved in hypoxic-ischemic brain injury of newborns^8^ and cardiovascular diseases.^49^

However, further research is needed to understand the mechanisms underlying our observations and their implications for preterm birth and related health outcomes. With our work we aim to provide a basis for future research in human infants which could include the comparison of healthy and ill preterm infants or further exploration of autophagy in various samples (e.g., including cell samples) collected during lung disease in infants.

One of the major strengths of this study is the large sample size compared to previous autophagy studies in preterm birth. Adjusting for risk factors is an important aspect of this study, enabled by the large sample size. However, missing data on preeclampsia, gestational diabetes and antibiotic use for some participants could limit the conclusions that can be drawn. Nevertheless, we included these risk factors in the sensitivity analysis. Another limitation is the uneven distribution of preterm infants between the centers, which we have considered by adjusting for center. The fact that the mechanism by which autophagy markers are detectable in cord blood is not fully understood is another limitation in our interpretation of these novel findings. The possible involvement of EVs^41,42^ and our analyses including leukocyte count and total protein level (Supplementary Tables S12 and S13) are helpful in exploring this issue, but further research is needed to better understand the mechanisms involved. Since there was no consistent and string association between leukocyte count or total protein level and autophagy marker levels, the representation of the marker levels per fixed plasma volume seemed appropriate for our study. Furthermore, due to the many clinically-related disturbing factors for blood sampling due to the birth circumstances, in this real-life setting there was heterogeneity in sampling procedures which we addressed by exclusion of incomparable samples and by adjusting for methodological factors. We cannot fully explain the biology of low p62 levels (below the detection limit). However, consistent with our data, low levels of p62 compared to LC3B levels were also described in serum in Fang et al.^44^ Furthermore, we excluded infants with p62 levels below the detection limit and observed even larger differences in p62 levels between preterm and term infants (Supplementary Table S10). In any case at this stage, the findings are observational and associative in nature. Nevertheless, our findings based on autophagy markers in cord blood plasma are consistent with cell and placental tissue models of premature birth.^33,34,39^

In conclusion, this study demonstrates differential levels of key autophagy markers in preterm compared to term infants and an association with gestational age. This adds to the knowledge of the sparsely studied field of autophagy mechanisms in preterm infants. Furthermore, it supports the hypothesis from human and animal cell and tissue models, that autophagy might be a link between impaired oxidative stress response, preterm birth, impaired lung development and higher susceptibility to respiratory morbidity in preterm infants.

## Supplementary Information

Supplementary Material

## Data Availability

The datasets generated during and/or analyzed during the current study are available from the corresponding author on reasonable request.
