# Supplementary material for: Differences in autophagy marker levels at birth in preterm vs. term infants
Source: Pediatr Res. 2024 May 29;96(5):1299–305. doi: 10.1038/s41390-024-03273-6 (PMC11521993; doi:10.1038/s41390-024-03273-6)
Supplement: Supplementary file 1 — Supplementary Material [file 41390_2024_3273_MOESM1_ESM.pdf]

Supplementary Material

Differences in autophagy marker levels at birth in preterm vs. term infants

Noëmi Künstle[1,2], Olga Gorlanova[1], Andrea Marten[1], Loretta Müller[2], Pawan Sharma[3], Martin Röösli[4], Pablo Sinues[1,5], Primo Schär[6], David Schürmann[6], Céline Rüttimann[1,2], Carla Rebeca Da Silva Sena[1,2,7], Uri Nahum[1,8], Jakob Usemann[1,2], Ruth Steinberg[1,2], Sophie Yammine[2], Sven Schulzke[1], Philipp Latzin[2], Urs Frey[1] on behalf of the BILD study group

[1]University Children's Hospital Basel UKBB, University of Basel, Basel, Switzerland

[2]Division of Pediatric Respiratory Medicine and Allergology, Department of Pediatrics, Inselspital, Bern University Hospital, University of Bern, Bern, Switzerland

[3]Center for Translational Medicine, Division of Pulmonary, Allergy and Critical Care Medicine, Jane & Leonard Korman Respiratory Institute, Sidney Kimmel Medical College, Thomas Jefferson University, Philadelphia, Pennsylvania

[4]Swiss Tropical and Public Health Institute, Allschwil, Switzerland and University of Basel, Basel, Switzerland

[5]Department of Biomedical Engineering, University of Basel, Allschwil, Switzerland

[6]Department of Biomedicine, University of Basel, Basel, Switzerland

[7]Priority Research Centre GrowUpWell® and Hunter Medical Research Institute, University of Newcastle, Newcastle, NSW, Australia

[8]Institute for Medical Engineering and Medical Informatics, University of Applied Sciences and Arts Northwestern Switzerland, Muttenz, Switzerland

Methods

Study design and population

The Basel-Bern Infant Lung Development (BILD) birth cohort is a prospective study established in 1999 in Bern and 2011 in Basel, both in Switzerland (www.bild-cohort.ch).[1,2] The aim of the ongoing study is to understand lung development and lung growth, and to identify factors (e.g., genetics and environmental factors such as air pollution) that have an influence on respiratory symptoms, lung functional outcome and lung diseases such as asthma. More details about the study protocol have been previously described.[1,2] In this study, infants born between April 1999 and February 2019 were included. Additional exclusion criteria were unavailable cord blood plasma (e.g., due to collapsing umbilical cord or storage by the parents for other purposes), cord blood donation due to differences in sampling procedure, missing clinical or sociodemographic data and time from blood collection to processing >3 days or insufficiently documented sampling procedure. This resulted in a study population of 64 preterm and 453 term infants.

Written informed consent was obtained from the parents. The study was approved by the Ethics Committee of Northwestern and Central Switzerland (EKNZ, Basel, Switzerland) and the Bernese Cantonal Ethics Research Committee (KEK, Bern, Switzerland).

Outcome assessment

Cord blood, which contains fetal blood, was collected after delivery by midwives and processed by our study nurses and laboratory assistants. In the case of postnatal recruitment, the routinely collected cord blood was used if it was no longer needed for clinical purposes. The cord blood was centrifuged (1811g, 6min, room temperature) to obtain the blood plasma and stored at -80°C until further processing. In a test round, random samples were diluted as suggested by the manufacturer. Dilution for the final analysis was then adjusted depending on the result of the test round. For each sample, 100µl of undiluted plasma was used for Beclin-1 and p62,

100µl of 1:2 diluted plasma of LC3B and 100µl of 1:1000 or 1:2000 diluted plasma for SIRT1. Samples were defrosted and aliquoted into 96-well plates, containing 80 plasma samples each and stored at -80°C again until analyses. To each 96-well plate 8x2 standard series were added. The manufacturer's protocol was strictly followed and detection limits reported by the manufacturer were used for each marker. The analysis was performed with BECN1 (Beclin-1) ELISA Kits (AVIVA Systems Biology, San Diego, CA, Cat#OKEH04415), MAP1LC3B ELISA Kits (AVIVA Systems Biology, San Diego, CA, Cat#OKEH01676), p62 ELISA Kits (ENZO Life Sciences, Farmingdale, NY, Cat#ADI-900-212), and SIRT1 ELISA Kits (AVIVA Systems Biology, San Diego, CA, Cat#OKEH01724).

Leukocytes and total protein

Leukocytes were analyzed by the hematological laboratory of the two study centers in cord blood within 12 hours after birth.

Total protein level was measured in cord blood plasma in a subgroup of 19 term infants. Analysis was performed with the Quick Start™ Bradford Protein Assay Kit 2 (Bio-Rad Laboratories, Inc., CA, #5000202).

Risk factors

Information on possible risk factors was taken from birth records and interviews with standardized questionnaires. The most important risk factors were small for gestational age (SGA, defined as birth weight below 10th percentile), mode of delivery (Caesarean section/vaginal delivery), sex (male/female), birth order (non-firstborn/firstborn) and maternal smoking during pregnancy (yes/no). Since the clinical assessment of intrauterine growth retardation (IUGR) is observer-dependent, SGA was used as a quantitative, well-defined proxy. Z-scores and percentiles for birth weight were calculated with the Fenton growth chart.[3] The same birth order was given to twins or triplets.

Statistical analysis

We compared autophagy marker levels between preterm and term infants using Tobit regression models to account for the left-censoring because of data below the limit of detection, adjusting for risk factors.

Anthropometric information was compared between centers. Only term infants were compared because preterm infants were almost only recruited in Basel. To examine differences in characteristics between centers, we compared the groups using the Mann–Whitney U test for continuous variables and Pearson's χ^2 test or Fisher's exact test for categorical variables.

Supplementary sensitivity analysis: We additionally adjusted the main model for maternal age at birth, because SIRT1 has been described to be downregulated in advanced maternal age placentas.[4] To prevent overadjustment we did not include maternal age from the beginning, since it is associated with birth order (non-firstborn/firstborn). Furthermore, to correct for the fact that many preterm infants were twins or triplets and experienced the same environment during pregnancy, we clustered for family ID in a multilevel mixed-effects Tobit regression (twins or triplets had the same family ID). Twins or triplets were designated as such only if both (or at least two) infants were included in the analyzed population.

Autophagy markers in plasma: There is limited knowledge on how the intracellular autophagy markers enter into plasma. To test if cell death could be a main mechanism, we calculated Spearman's correlation coefficient between leukocyte count and autophagy marker levels. Only term infants were included for these analyses. We excluded infants with a leukocyte count $<3x10^9$L or hemoglobin <100g/L (n=4) which resulted in 293 infants in total. We hypothesized that a decreasing leukocyte count in combination with an increase in autophagy marker levels would indicate the release of intracellular markers through cell death. Furthermore, we measured total protein level in a subgroup of 19 term infants to assess if autophagy marker

levels are systematically dependent on total protein level. To analyze this, we calculated Spearman's correlation coefficient between total protein level and autophagy marker levels.

Data analysis was performed in Stata 16 (StataCorp, College Station, TX) and R version 4.3.2 (R Foundation, Vienna, Austria, https://www.r-project.org/).

Results

Significant center differences in term infants were found in respect to Apgar score at 5 minutes and antibiotic use during the last twelve weeks of pregnancy (**Table S1**).

Supplementary sensitivity analysis: After the additional adjustment for maternal age, levels of p62 remained significantly higher and levels of SIRT1 significantly lower in preterm infants (**Table S5**). Because inclusion of family ID as a random effect did not substantially change results, we decided to use the simpler model without family ID.

Autophagy markers in plasma: There was no negative correlation between leukocyte count and autophagy marker levels, and we detected only a weak positive correlation with SIRT1 (r_s= 0.25) (**Table S12**). Therefore, we interpreted this to mean that cell death is not the main reason for the release of autophagy markers into plasma.

Looking at the correlation between total protein and autophagy marker levels, we found no correlation with autophagy marker levels (**Table S13**) which is why we assume that autophagy marker levels are not systematically dependent on total protein level. Therefore, the representation of the marker levels per fixed plasma volume seemed appropriate for our study.

Tables and figures

Table S1: Study population characteristics for term infants in Basel and Bern

	**Total** n=453	**Basel** n=65	**Bern** n=388	**p-value**[a]
Gestational age at birth, weeks	39.78 (1.12)	39.67 (1.10)	39.79 (1.13)	0.363
Sex, male	232 (51%)	31 (48%)	201 (52%)	0.539
Mode of delivery, Caesarean section	88 (19%)	17 (26%)	71 (18%)	0.138
Weight at birth, z-score	-0.17 (0.85)	-0.27 (0.89)	-0.15 (0.85)	0.368
Small for gestational age	41 (9%)	8 (12%)	33 (9%)	0.323
Apgar, 5 minutes[b]	9.17 (0.87)	9.58 (0.63)	9.10 (0.88)	<0.001
Birth order, non-firstborn	245 (54%)	35 (54%)	210 (54%)	0.967
Twin[c]	4 (1%)	2 (3%)	2 (1%)	0.100
Maternal smoking during pregnancy	26 (6%)	4 (6%)	22 (6%)	0.778
Antibiotics during the last 12 weeks of pregnancy[d]	36 (8%)	14 (22%)	22 (6%)	<0.001
Chorioamnionitis[e]	5 (1%)	1 (2%)	4 (1%)	0.538
Preeclampsia[f]	0 (0%)	0 (0%)		
Gestational diabetes[f]	4 (1%)	4 (6%)		
Maternal age at birth, years	32.66 (4.23)	33.34 (4.02)	32.54 (4.26)	0.176

Data are presented as mean±SD or n (%).
[a]P-values were obtained using the Mann–Whitney U test, Pearson's χ^2 test or Fisher's exact test.
[b]Data available for n=452 (Basel, n=65; Bern, n=387).
[c]Only considered if both siblings included in study.
[d]Data available for n=410 (Basel, n=65; Bern, n=345).
[e]Data available for n=442 (Basel, n=63; Bern, n=379).
[f]Data available for n=54 (Basel, n=54; Bern, n=0).

Table S2: Cord blood autophagy marker levels and detection rates

	Total		
	**Detection limit**	**Median (IQR)**	**Detection rate**
Beclin-1, ng/ml	0.1	0.50 (0.36-0.65)	100%
LC3B, ng/ml	0.078	2.03 (1.11-2.91)	99%
p62, ng/ml	0.1	0.29 (0.01-0.72)	66%
SIRT1, µg/ml	0.000032	2.20 (1.10-3.50)	99%

Definition of abbreviations: IQR=interquartile range.

Table S3: Association between gestational age and cord blood autophagy marker levels

	Coef	95% CI	p-value	Adj. p-value
Beclin-1, ng/ml	-0.002	-0.01 to 0.01	0.568	0.757
LC3B, ng/ml	0.002	-0.02 to 0.03	0.860	0.860
p62, ng/ml			0.009	0.018
SIRT1, µg/ml	0.10	0.07 to 0.13	5.57×10^{-10}	2.23×10^{-9}

Definition of abbreviations: Coef=coefficient; CI=confidence interval; Adj.=adjusted.
Tobit regression model was used for Beclin-1, LC3B and SIRT1. Coefficient is shown for preterm infants in comparison to term infants (reference group). For p62 generalized additive Tobit model (GAM) was used because of non-linear association and the p-value for the smoothing function is shown. All models were adjusted for small for gestational age (SGA), mode of delivery, sex, birth order, maternal smoking during pregnancy, time until processing, temporary fridge storage and center. Results are presented in log2-transformed level. Adjusted p-values (n=4) were calculated using the Benjamini–Hochberg method.

Table S4: Correlations between autophagy markers

	Total	Preterm			Term		
	r_s	r_s	95% CI	Adj. p-value	r_s	95% CI	Adj. p-value
Beclin-1, LC3B	0.70	0.56	0.37 to 0.71	$2.18x10^{-6}$	0.72	0.67 to 0.76	$2.14x10^{-71}$
p62, LC3B	-0.52	-0.56	-0.71 to -0.37	$2.18x10^{-6}$	-0.49	-0.56 to -0.42	$1.13x10^{-28}$
SIRT1, LC3B	-0.05	-0.06	-0.30 to 0.19	0.658	-0.13	-0.22 to 0.04	0.006

Definition of abbreviations: r_s=Spearman's correlation coefficient; CI=confidence interval; Adj.=adjusted. Adjusted p-values (n=6) were calculated using the Benjamini–Hochberg method.

Table S5: Association between preterm birth and cord blood autophagy marker levels in comparison to term infants with additional adjustment for maternal age

	Coef	95% CI	p-value
Beclin-1, ng/ml	0.03	-0.03 to 0.10	0.334
LC3B, ng/ml	-0.03	-0.24 to 0.18	0.783
p62, ng/ml	0.37	0.05 to 0.69	0.025
SIRT1, µg/ml	-0.53	-0.77 to -0.29	<0.001

Definition of abbreviations: Coef=coefficient; CI=confidence interval.
Tobit regression model was used, adjusted for small for gestational age (SGA), mode of delivery, sex, birth order, maternal smoking during pregnancy, time until processing, temporary fridge storage center and maternal age. Coefficient is shown for preterm infants in comparison to term infants (reference group). Results are presented in log2-transformed level.

Table S6: Association between preterm birth and cord blood autophagy marker levels in comparison to term infants with additional adjustment for preeclampsia (n=111)

	Coef	95% CI	p-value
Beclin-1, ng/ml	-0.02	-0.11 to 0.07	0.633
LC3B, ng/ml	-0.22	-0.55 to 0.11	0.184
p62, ng/ml	0.72	0.20 to 1.25	0.008
SIRT1, µg/ml	-0.49	-0.81 to -0.16	0.004

Definition of abbreviations: Coef=coefficient; CI=confidence interval.
Tobit regression model was used, adjusted for small for gestational age (SGA), mode of delivery, sex, birth order, maternal smoking during pregnancy, time until processing, temporary fridge storage and preeclampsia. Coefficient is shown for preterm infants in comparison to term infants (reference group). Results are presented in log2-transformed level.

Table S7: Association between preterm birth and cord blood autophagy marker levels in comparison to term infants with additional adjustment for gestational diabetes (n=111)

	**Coef**	**95% CI**	**p-value**
Beclin-1, ng/ml	-0.02	-0.11 to 0.07	0.605
LC3B, ng/ml	-0.21	-0.52 to 0.11	0.198
p62, ng/ml	0.77	0.25 to 1.29	0.004
SIRT1, µg/ml	-0.47	-0.78 to -0.15	0.004

Definition of abbreviations: Coef=coefficient; CI=confidence interval.
Tobit regression model was used, adjusted for small for gestational age (SGA), mode of delivery, sex, birth order, maternal smoking during pregnancy, time until processing, temporary fridge storage and gestational diabetes. Coefficient is shown for preterm infants in comparison to term infants (reference group). Results are presented in log2-transformed level.

Table S8: Association between preterm birth and cord blood autophagy marker levels in comparison to term infants with additional adjustment for antibiotic use during the last twelve weeks of pregnancy (n=472)

	**Coef**	**95% CI**	**p-value**
Beclin-1, ng/ml	0.03	-0.03 to 0.10	0.331
LC3B, ng/ml	-0.04	-0.25 to 0.18	0.744
p62, ng/ml	0.38	0.06 to 0.70	0.019
SIRT1, µg/ml	-0.55	-0.79 to -0.30	<0.001

Definition of abbreviations: Coef=coefficient; CI=confidence interval.
Tobit regression model was used, adjusted for small for gestational age (SGA), mode of delivery, sex, birth order, maternal smoking during pregnancy, time until processing, temporary fridge storage, center and antibiotic use during the last twelve weeks of pregnancy. Coefficient is shown for preterm infants in comparison to term infants (reference group). Results are presented in log2-transformed level.

Table S9: Association between preterm birth and cord blood autophagy marker levels in comparison to term infants with additional adjustment for chorioamnionitis (n=504)

	**Coef**	**95% CI**	**p-value**
Beclin-1, ng/ml	0.02	-0.05 to 0.09	0.554
LC3B, ng/ml	-0.03	-0.25 to 0.19	0.767
p62, ng/ml	0.42	0.09 to 0.75	0.013
SIRT1, µg/ml	-0.52	-0.77 to -0.27	<0.001

Definition of abbreviations: Coef=coefficient; CI=confidence interval.
Tobit regression model was used, adjusted for small for gestational age (SGA), mode of delivery, sex, birth order, maternal smoking during pregnancy, time until processing, temporary fridge storage, center and chorioamnionitis. Coefficient is shown for preterm infants in comparison to term infants (reference group). Results are presented in log2-transformed level.

Table S10: Association between preterm birth and cord blood autophagy marker levels in comparison to term infants after exclusion of infants with p62 levels below the detection limit (n=341)

	**Coef**	**95% CI**	**p-value**
p62, ng/ml	0.42	0.12 to 0.72	0.007

Definition of abbreviations: Coef=coefficient; CI=confidence interval.
Tobit regression model was used, adjusted for small for gestational age (SGA), mode of delivery, sex, birth order, maternal smoking during pregnancy, time until processing, temporary fridge storage and center.
Coefficient is shown for preterm infants in comparison to term infants (reference group). Results are presented in log2-transformed level.

Table S11: Association between preterm birth and cord blood autophagy marker levels in comparison to term infants in a sex stratified analysis

	Female infants			Male infants			
	**Coef**	**95% CI**	**p-value**	**Coef**	**95% CI**	**p-value**	**p-value for interaction**
Beclin-1, ng/ml	0.01	-0.08 to 0.10	0.820	0.05	-0.05 to 0.14	0.325	0.826
LC3B, ng/ml	-0.12	-0.43 to 0.20	0.473	0.04	-0.25 to 0.32	0.788	0.765
p62, ng/ml	0.44	-0.04 to 0.92	0.074	0.29	-0.14 to 0.72	0.189	0.363
SIRT1, µg/ml	-0.51	-0.86 to -0.16	0.005	-0.54	-0.87 to -0.21	0.001	0.224

Definition of abbreviations: Coef=coefficient; CI=confidence interval.
Tobit regression model was used and stratified by sex, adjusted for small for gestational age (SGA), mode of delivery, birth order, maternal smoking during pregnancy, time until processing, temporary fridge storage and center. Coefficient is shown for preterm infants in comparison to term infants (reference group). Results are presented in log2-transformed level. The p-value for the interaction was calculated by including a multiplicative interaction term between prematurity and sex in the adjusted model which included all infants.

Table S12: Correlations between autophagy markers and leukocyte count in term infants (n=293)

	r_s	p-value
Beclin-1, ng/ml	0.07	0.222
LC3B, ng/ml	0.08	0.197
p62, ng/ml	0.11	0.060
SIRT1, µg/ml	0.25	<0.001

Definition of abbreviations: r_s=Spearman's correlation coefficient.

Table S13: Correlations between autophagy markers and total protein in term infants (n=19)

	r_s	p-value
Beclin-1, ng/ml	0.20	0.401
LC3B, ng/ml	-0.10	0.695
p62, ng/ml	0.24	0.328
SIRT1, µg/ml	0.37	0.115

Definition of abbreviations: r_s=Spearman's correlation coefficient.

Table S14: Correlation between autophagy marker levels and storage duration (time from storage to analysis)

	r_s
Beclin-1, ng/ml	0.10
LC3B, ng/ml	0.15
p62, ng/ml	-0.11
SIRT1, μg/ml	0.11

Definition of abbreviations: r_s=Spearman's correlation coefficient.

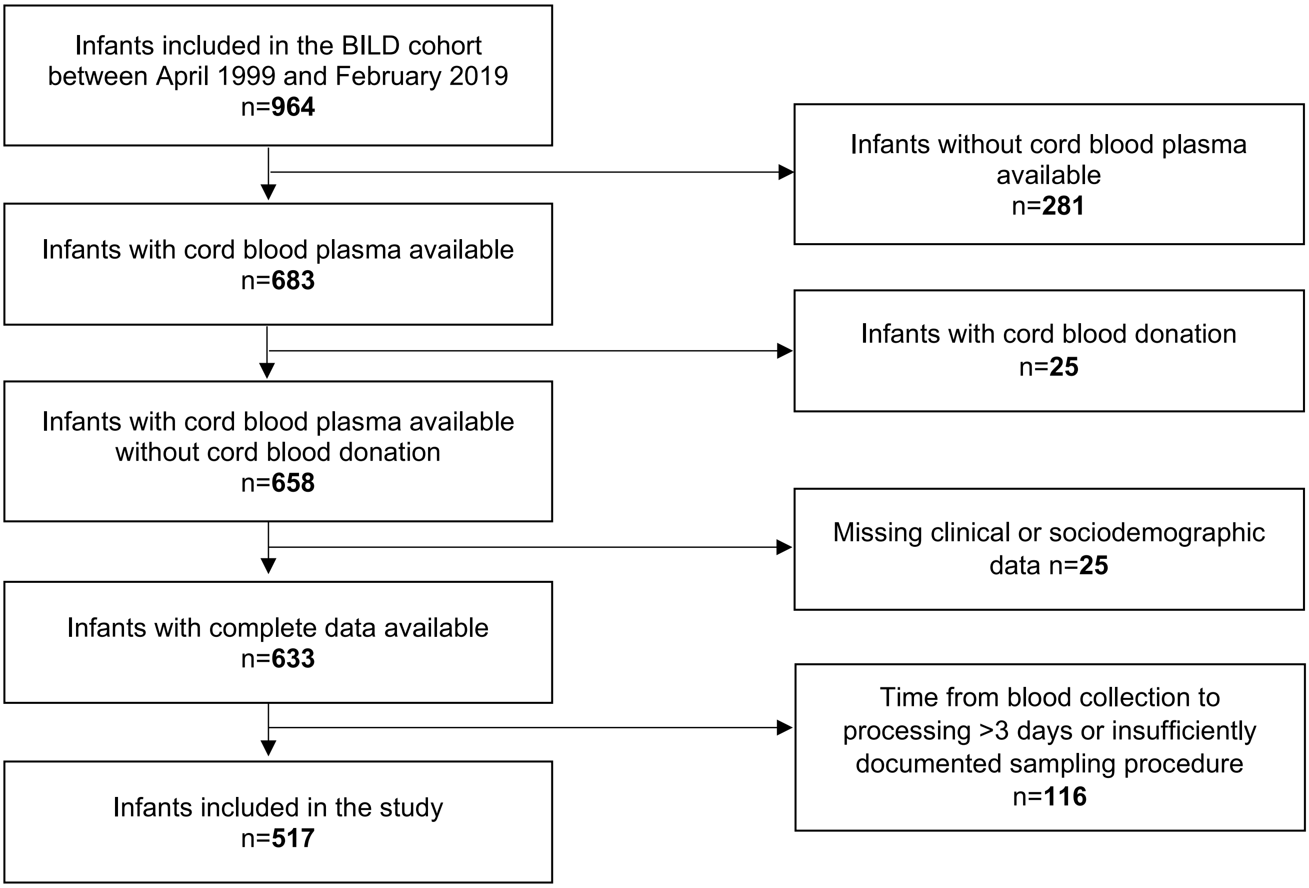

Fig. S1: Flow chart of study population.

References

1 Fuchs, O., Latzin, P., Kuehni, C. E. & Frey, U. Cohort profile: the Bern infant lung development cohort. *Int. J. Epidemiol.* **41**, 366-376 (2012).

2 Salem, Y. et al. Cohort Profile Update: The Bern Basel Infant Lung Development Cohort. *Int. J. Epidemiol.*, dyad164 (2023).

3 Fenton, T. R. & Kim, J. H. A systematic review and meta-analysis to revise the Fenton growth chart for preterm infants. *BMC Pediatr.* **13**, 1-13 (2013).

4 Xiong, L. et al. Advanced Maternal Age-associated SIRT1 Deficiency Compromises Trophoblast Epithelial− Mesenchymal Transition through an Increase in Vimentin Acetylation. *Aging Cell* **20**, e13491 (2021).
